# Progression of m^6^A in the tumor microenvironment: hypoxia, immune and metabolic reprogramming

**DOI:** 10.1038/s41420-024-02092-2

**Published:** 2024-07-20

**Authors:** Xuan Han, Yu Zhu, Juan Ke, Yufeng Zhai, Min Huang, Xin Zhang, Hongjie He, Xiaojing Zhang, Xuehong Zhao, Kaikai Guo, Xianglin Li, Zhongyu Han, Yanming Zhang

**Affiliations:** 1 First Clinical College of Changzhi Medical College, Changzhi, China; 2 Linfen Central Hospital, Linfen, China; 3 School of Medicine and Life Sciences, Chengdu University of Traditional Chinese Medicine, Chengdu, China

**Keywords:** Cancer microenvironment, Immune evasion, Apoptosis, DNA metabolism

## Abstract

Recently, N6-methyladenosine (m^6^A) has aroused widespread discussion in the scientific community as a mode of RNA modification. m^6^A comprises writers, erasers, and readers, which regulates RNA production, nuclear export, and translation and is very important for human health. A large number of studies have found that the regulation of m^6^A is closely related to the occurrence and invasion of tumors, while the homeostasis and function of the tumor microenvironment (TME) determine the occurrence and development of tumors to some extent. TME is composed of a variety of immune cells (T cells, B cells, etc.) and nonimmune cells (tumor-associated mesenchymal stem cells (TA-MSCs), cancer-associated fibroblasts (CAFs), etc.). Current studies suggest that m^6^A is involved in regulating the function of various cells in the TME, thereby affecting tumor progression. In this manuscript, we present the composition of m^6^A and TME, the relationship between m^6^A methylation and characteristic changes in TME, the role of m^6^A methylation in TME, and potential therapeutic strategies to provide new perspectives for better treatment of tumors in clinical work.

## Facts

- m^6^A is the most common epigenetic modification in eukaryotic mRNAs.
- Writes, erasers, and readers together comprise m^6^A and are involved in regulating RNA production, nuclear export, and translation and degradation, which have important implications and implications for many pathophysiological processes, including tumors.
- TME is an environment that affects the survival of tumor cells and is characterized by hypoxia, acid accumulation, and immunosuppression.
- m^6^A can affect hypoxia, metabolic reprogramming, immunosuppression and then affect the occurrence and development of tumors in TME.

## Open Questions

- How do m^6^A-related proteins play a role in each associated tumor and what are their effects?
- Interassociation of various cells in the TME with m^6^A and tumors in relation to tumor progression and treatment efficacy.
- Whether m^6^A could be a potential target for cancer therapy by affecting the pre-metastatic niche and metastatic niche have an impact on the biological characteristics of tumor malignancy?

## Introduction

**Fig. 1:**
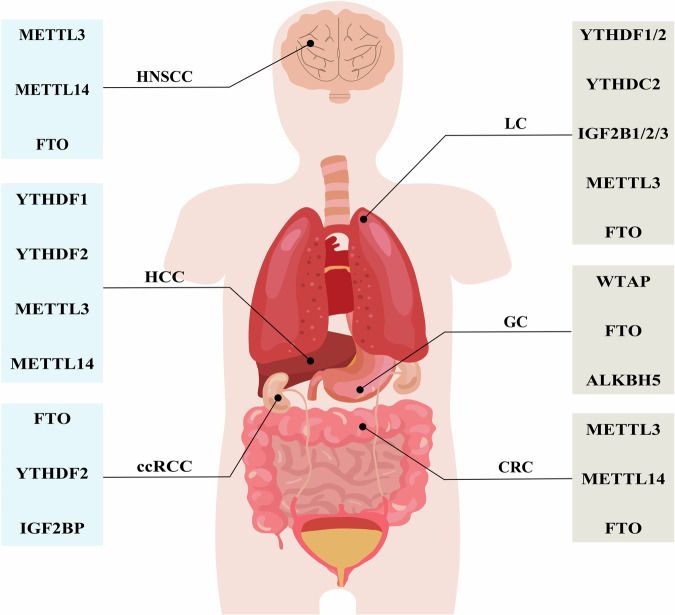
Effect of m^6^A methylation in tumors. CRC colorectal cancer, ccRCC renal clear cell carcinoma, GC gastric cancer, HCC hepatocellular carcinoma, HNSCC head and neck squamous cell carcinoma, LC lung cancer.

The concept of RNA modification was first introduced 50 years ago, and more than 100 types have now been identified [1–3]. With advances in technology, N6-methyladenosine (m^6^A) can be found as a modification in a variety of RNA types [4]. Numerous studies have shown that m^6^A is the most common type of base modification and occurs predominantly in mRNA [5]. In recent years, the structure and function of m^6^A methylation have been gradually understood. m^6^A methylation occurs at the N6 position of adenosine, mostly distributed in coding sequences, the 3′ ends of transcripts, and stop codons [6, 7]. m^6^A methylation is a dynamically variable process that is mediated and regulated by three protein factors: writes, erasers, and readers [8]. These three regulators can affect RNA splicing, export, translation, and degradation and participate in a variety of pathophysiology activities including neoplasms [9–11]. Numerous studies have found that m^6^A RNA modifications have been detected in a wide range of tumors and the aberrant modification is important for tumorigenesis and oncogene expression [12–14] (Fig. 1 and Table 1). m^6^A methylation drives tumor progression by regulating hypoxia, metabolic reprogramming, immunosuppressive properties, and acidic environment in the tumor microenvironment (TME) [12, 15, 16].

**Table 1:** Tumors associated with m^6^A methylation.

Tumor type	m^6^A regulator	Mechanism and effect	References
HNSCC	ALKBH5	Inhibiting ALKBH5/RIG-I/IFNα axis and promoting tumor development	[142]
NPC	FTO	Suppressing immune cell levels and reducing survival	[177]
OSCC	FTO	Increasing expression of PD-L1 transcripts and promoting tumor development	[279]
GBM	METTL3	Promoting maturation of SLC7A11 mRNA and suppressing ferroptosis	[280]
	ALKBH5	Promoting expression level of FOXM1 and promoting tumor proliferation and metastasis	[281]
EC	FTO	Promoting expression of HSD17B11 and facilitating tumor progression	[103]
BC	METTL3/ IGF2BP3	Stabilizing PD-L1 mRNA via METTL3/IGF2BP3 axis and promoting tumor immune escape	[137]
	YTHDF1	Inhibiting PKM2 expression and promoting tumor growth and metastasis	[85]
	YTHDF3	Enhancing expression of m^6^A-enriched gene transcripts and facilitating malignancy	[282]
	WTAP	Increasing ENO1 mRNA stability and promoting tumor development	[283]
LC	METTL3	Reducing gene expression of HMBOX1 and promoting tumor proliferation	[284]
	METTL3/ ALKBH5	Enhancing ENO1 translation and promoting tumorigenesis	[285]
	YTHDF2	Activating mTOR/AKT and promoting tumorigenesis and invasion	[68]
	YTHDC2	Inhibiting SLC3A2 expression via YTHDC2/HOXA13/SLC3A2 axis and inducing ferroptosis	[286]
	FTO	Inhibiting Wnt/β-catenin/FTO/c-Myc and suppressing tumor progression	[287]
	IGF2BP1	Inhibiting Netrin-1 expression and suppressing tumor progression	[288]
HCC	METTL3	Stabilizing lncRNA LINC00958 and facilitating tumor development	[96]
	METTL14	Increasing MIR155HG stability through METTL14/MIR155HG/PD-L1 axis and promoting tumor immune escape	
	YTHDF1/ METTL3	Enhancing EMT by mediating Snail mRNA translation and promoting tumor metastasis	[289]
	YTHDF2	Inhibiting IL-11 and SERPINE2 RNA degradation and inducing tumorigenesis	[290]
	WTAP	Blocking cell cycle transition and facilitating tumor progression	[291]
GC	METTL3	Accelerating m^6^A methylation of HDGF mRNA and promoting tumor development	[292]
	METTL14	Suppressing LHPP expression and promoting tumor progression	[293]
	WATP	Promoting HK2 mRNA stability and facilitating tumor progression	[90]
PC	ALKBH5	Aggregating CD8^+^ and CD4^+^ T cells and promoting tumor evade immune surveillance	[294]
PDAC	YTHDF3	Promoting miR-30d and inhibiting RUNX1 via YTHDC1/miR-30d/RUNX1axis and inhibiting tumorigenesis	[88]
CRC	METTL3	Enhancing the stability of HK2 and GLUT1 transcripts and facilitating tumor progression	[224]
	METTL14	Affecting normal function of CD8^+^ T cells and facilitating malignancy	[295]
	YTHDF1	Enhancing cisplatin resistance through metabolic reprogramming and suppressing tumor cell death	[114]
	IGF2BP2	Increasing MYC mRNA stability via the LINRIS-IGF2BP2-MYC axis and promoting tumor development	[235]
	FTO	Reducing MTA1 mRNA stability and suppressing tumor progression and metastasis	[73]
BCa	METTL3	Stabilizing PD-L1 mRNA via JNK/METTL3/PD-L1 axis and promoting tumor immune escape	[296]
	ALKBH5	Inhibiting stability of CK2α mRNA and inhibiting cisplatin resistance	[297]
RCC	METTL14	Inhibiting BPTF mRNA stability and suppressing tumor metastasis	[298]
	IGF2BP1	Stabilizing mRNA and promoting G1/S cell cycle transition and enhancing tumor growth	[299]
	FTO	Inhibiting YTHDF2-dependent Unc-51-like kinase 1 mRNA decay and promoting tumor development	[300]
ccRCC	FTO	Increasing expression of SLC1A5 and facilitating tumor progression	[115]
OC	METTL3	Promoting maturation and function of NK cells and improving immune function	[170]
	ALKBH5	Increasing EGFR-PIK3CA-AKT-mTOR activity and promoting tumor progression	[301]
CC	METTL3/ YTHDF1	Stabilizing HK2 mRNA through YTHDF1 and promoting tumor development	[86]
PCa	YTHDF2	Binding to NKX3-1 and LHPP and facilitating tumor growth	[302]
Leukemia	METTL3	Affecting epithelial-mesenchymal transition in cancer cells by altering TGFβ1 expression and secretion and promoting tumor progression	[303]
	YTHDF2	Inhibiting m^6^A expression via AML1/ETO-HIF-1α-YTHDF2 axis and accelerating tumor growth	[304]
Melanoma	FTO	Inhibiting CD8^+^ T cells function by promoting expression of transcription factors c-Jun, JunB, and C/EBPβ and facilitating immune evasion	[92]
	YTHDF2	Promoting maturation and function of NK cells and improving immune function	[169]

*m*^*6*^*A* N6-methyladenosine, *HNSCC* head and neck squamous cell carcinoma, *NPC* nasopharyngeal carcinoma, *OSCC* oral squamous cell carcinoma, *GBM* glioblastoma, *EC* esophageal cancer, *BC* breast cancer, *LC* lung cancer, *HCC* hepatocellular carcinoma, *GC* gastric cancer, *PC* pancreatic cancer, *PDAC* pancreatic ductal adenocarcinoma, *CRC* colorectal cancer, *BCa* bladder cancer, *RCC* renal carcinoma, *ccRCC* renal clear cell carcinoma, *OC* ovarian Cancer, *CC* cervical cancer, *PCa* prostatic carcinoma.

TME is the environment for tumor-associated cell survival, mainly consisting of various types of cells such as tumor cells, immune cells, inflammatory cells, immunosuppressive cells, vascular cells, and surrounding biomolecules [17]. In recent years, with the understanding of TME, the potential relationship between tumors and TME has received increasing attention. Hypoxia, metabolic reprogramming, acidic environment, and immunosuppression are specific features of TME [18]. These components are involved in regulating a variety of pathological processes including tumors [19]. It has been found that TME interacts with tumor cells in many aspects, including tumor growth, differentiation, invasion, and resistance [20]. TME is a complex system and targeting the tumor-associated pathways within it can inhibit the activity of tumor-associated cells and enhance immune response [21, 22].

Through understanding m^6^A m methylation and TME, we found that they have multiple implications for tumor development. Here, we summarize the role of m^6^A methylation in TME by elaborating the relationship between m^6^A methylation and characteristic changes in TME and methylation programs, supplement potential clinical applications, and improve new perspectives for better diagnosis and treatment of tumors in future clinical work.

## m^6^A modification

### m^6^A writers—methyltransferase

m^6^A writers are methyltransferases whose main function is to mount methyl groups and add m^6^A methylation sites. m^6^A writers typically function as multifunctional subunit complexes, including methyltransferase-like 3 (METTL3), methyltransferase-like 5 (METTL5), methyltransferase-like 14 (METTL14), methyltransferase-like 16 (METTL16), William’s tumor 1-associated protein (WTAP), RNA-binding motif protein 15/15B (RBM15/15B), vir-like m^6^A methyltransferase-associated (VIRMA/KIAA1429), phosphorylated CTD-interacting factor 1 (PCIF1), and zinc finger CCCH-type containing 4/13 (ZC3H4, ZC3H13). These components vary in function, METTL3, METTL14, and WTAP are implicated in the formation and initiation of m^6^A [23], with METTL3 having catalytic capacity, while METTL14 can activate and promote METTL3 viability, and WTAP localizes METTL3 and METTL14 to the nuclear center [24, 25]. METTL16 not only can regulate the expression of enzyme activity but also participates in the m^6^A modification of other types of RNA [26]. KIAA1429 and RBM15/15B have been reported to be important components of WTAP-related effects, involved in the clustering of the complex at specific positions [27, 28]. PCIF1 replication methylates adenine at the 5 ‘end of mRNA for m^6^A and regulates gene expression [29]. In addition, it was found that METTL5 and ZCCH4 can add the m^6^A to the 18 S and 28 S ribosomal RNAs [30, 31].

### m^6^A erasers—demethylases

m^6^A erasers remove m^6^A methylation sites and mediate dynamic changes in m^6^A methylation. There is substantial evidence that the demethylation of m^6^A is catalyzed primarily by two enzymes, fat mass and obesity-associated protein (FTO) and alpha-ketoglutarate-dependent dioxygenase ALKB homolog 5 (ALKBH5) [32]. FTO has oxidative effects and is primarily responsible for removing the demethylation of m^6^A in the nucleus, particularly in mRNA [33–35]. FTO has been shown to promote the progression of several tumors including leukemia and melanoma [36, 37]. ALKBH5 is primarily involved in the demethylation process in the cytoplasm and can be directly catalyzed by co-binding to nuclear speckles [38]. Unlike FTO, ALKBH5 is not involved in other types of RNA modification and is the only m^6^A modulator. Hypoxia will cause ALKBH5 expression, which leads to the development of disease [39, 40]. Recently, ALKB homolog 3 (ALKBH3) has been identified in the m^6^A modification, which acts preferentially on tRNAs [41].

### m^6^A readers—reading proteins

m^6^A readers are responsible for reading m^6^A methylation sites, enabling RNA binding to proteins, activating regulatory pathways, and affecting RNA splicing, export, translation, and degradation [11]. Current studies have found that readers are mainly composed of YTH domain-containing proteins (YTHDF1/2/3, YTHDC1/2), IGF2 mRNA-binding proteins (IGF2BPs), and METTL3 in the cytoplasm. These proteins are known to play different biological roles at different sites. Among them, YTHDF1/2/3 regulates RNAs mainly in the cytoplasm [42, 43]. YTHDF1 promotes the translation of target RNAs upon binding to other substances [44]. YTHDF2 is accountable for RNA stability and selective degradation [12, 45]. YTHDF3 acts by affecting YTHDF1/2, which strengthens the translation of YTHDF1 and the degradation of YTHDF2 after binding [46]. YTHD1/2 works mostly in the nucleus. YTHDC1 is not only associated with the splicing of RNA but also has some effect on mediating chromosome silencing [47, 48]. YTHDC2 can mediate the translation and degradation of RNA [49]. IGF2B1/2/3 combined with the m^6^A CU sequence improves RNA stability and expression and has oncogenic effects [50]. Particularly, expression was decreased in lung cancer [51]. METTL3 is also methyltransferase, but in the cytosol, it is m^6^A readers and promotes RNA translation as well as YTHDF1 [52]. In addition, there are also several novel m^6^A readers with the ability to mediate m^6^A modifications, such as fragile X mental retardation protein (FMRP) and eukaryotic initiation factor 3 (eIF3) [53, 54].

## Relationship of m^6^A methylation and TME characteristics

### m^6^A methylation and TME hypoxia

**Fig. 2:**
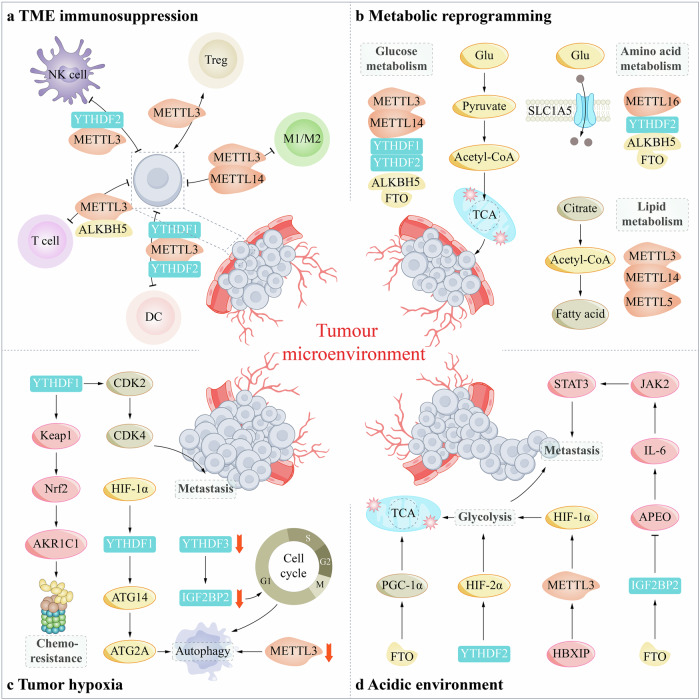
The tumor microenvironment (TME) is characterized by hypoxia, metabolic reprogramming, acidity, and immunosuppression. HIF impacts tumor cells through m^6^A methylation modification under hypoxia (**c**). There is a potential correlation between hypoxia and metabolic reprogramming, and the hypoxic environment promotes the effects of metabolic reprogramming on tumors. In addition, glucose metabolism, amino acid metabolism, and lipid metabolism affect the TME in response to m^6^A methylation which in turn affects tumor development (**b**). The m^6^A-mediated metabolic dysregulation generates an acidic environment, and both hypoxia and acidic environments promote the formation of immunosuppressive TME (**d**), and the function of multiple immune cells is also affected to promote the immunosuppressive microenvironment (**a**), further supporting tumor growth.

Hypoxia refers to a state of lack of adequate oxygen supply to cells, tissues, and organs, and as an important regulator of TME, oxygen partial pressure below 10 mmHg can be considered hypoxic TME [55]. Many researchers have found that hypoxia is a common and persistent manifestation in many solid tumors and is able to affect a range of biological behaviors, treatment outcomes, and prognosis of tumors [56]. Notably, m^6^A methylation is also altered in hypoxic environments, altering the biological morphology and behavior of tumors, and tumor immunity, metabolism, etc. [57]. Hypoxia-inducible factor (HIF), as the main transcription factor activated by hypoxia, plays a series of roles by regulating the expression of genes related to tumor development and mediating the body’s response to hypoxia, such as affecting the function of immune cells, angiogenesis, EMT, proliferation and survival of tumor cells, invasion and metastasis, and treatment resistance [58–61] (Fig. 2 and Table 2). Under hypoxia, HIF is an active heterodimeric complex composed of four subunits, HIF-1α, HIF-2α, HIF-3α, and HIF-1β, of which the former two subunits play a major role in the hypoxic environment [62]. HIF-1 and HIF-2 are heterodimers composed of HIF-1α and HIF-1β subunits, HIF-2α and HIF-1β subunits, respectively, and both have important roles in a positive hypoxic environment [63, 64]. HIF-3, on the other hand, has multiple isoforms and is considered a negative regulator [65]. Numerous studies have shown that HIF-1α is associated with the progression of a variety of tumors. For example, HIF-1α was found to not only promote the glycolytic process but also enhance the expression of YTHDF1/2 in hepatocellular carcinoma (HCC) and lung squamous cell carcinoma (LUSC), respectively, leading to cancer cell spread [66–68]. Similarly, in gastric cancer(GC), HIF-1α can be reduced by knocking down IGF2BP, which in turn achieves the effect of controlling the malignant growth of cancer cells [69]. Notably, high expression of YTHDF2 under hypoxia instead retarded HCC progression [70]. Another study showed that HIF-2α is associated with pancreatic cancer, and its presence inhibits the efficacy of tumor therapy and causes drug resistance [71]. In the previous section, we presented FTO and ALKBH5, which, although both proteins belong to m^6^A erasers, have distinct functions in hypoxia. Studies have confirmed that low expression of FTO in a hypoxic environment accelerates the malignant progression of colorectal cancer (CRC), while high expression of ALKBH5 significantly promotes female malignant tumors [15, 72, 73]. This evidence suggests that FTO and ALKBH5 are carcinogenic and tumor suppressor factors, respectively, in hypoxia.

**Table 2:** HIF and their regulatory targets and effects on tumors.

HIF	Target gene	Effects on tumors	References
HIF-1	ANGPT2, VEGF, PGF, GLUT1, LDHA, PKM2, EPO, IGF2, TGFA, Vimentin, Snail, TCF3, CAR9, BNIP3, HK1/2, SLC2A1, SLC7A11, MCT4, MT2-MMP, LOX, CypA, PD-L1, CD133, ADAM10, NF-kB, CXCR4, LncRNA-BX111	Angiogenesis, autophagy, glycolysis, survival and proliferation, EMT, metabolic reprogramming, migration and invasion, immune evasion, treatment resistance, cell-ECM interactions, cancer stem cell-like properties	[305–325]
HIF-2	VEGF, GLUT1, NANOG, SERPINE2, IL-11, uPAR1, ITGA6, MMP14	Angiogenesis, glycolysis, survival and proliferation, long-term hypoxic response, EMT, vessel integrity, migration and invasion, cell-ECM interactions, invasion and metastasis, immune evasion	[305, 306, 326–331]
HIF-3	SQRDL, LC3C, REDD1	Tumorigenesis, apoptosis, negative regulator of HIF-1/2	[332–334]

*ANGPT1* angiopoietin 1, *VEGF* vascular endothelial growth factor, *PGF* transforming growth factor, *GLUT1* glucose transporter 1, *LDHA* lactate dehydrogenase A, *PKM2* pyruvate kinase M2, *EPO* erythropoietin, *IGF2* insulin-like growth factor 2, *TGFA* transforming growth factor α, *TCF3* transcription factor 3, *CAR9* carbonic anhydrase 9, *BNIP3* BCL2 interacting protein 3, *HK1/2* hexokinase 1 and 2, *SLC2A1* solute carrier family 2 member 1, *MCT4* monocarboxylate transporter 4, *MT2-MMP* membrane type-2 matrix metalloproteinase, *LOX* lysyl oxidase, *CypA* cyclophilin A, *ADAM10* a disintegrin and metalloproteinase domain 10, *NF-kB* nuclear factor kappa B, *CXCR4* chemokine receptor 4, *EMT* epithelial-mesenchymal transition, *SERPINE2* serpin family E member 2, IL-11 interleukin-11, *uPAR1* urokinase-type plasminogen activator receptor-1, *ITGA6* integrin subunit alpha 6, *MMP14* matrix metalloproteinase 14, *SQRDL* sulfide-quinone reductase-like protein, *LC3C* MAP1LC3C, *REDD1* regulated in development and DNA damage responses 1.

Studies to date have established that hypoxia and tumor development are mutually reinforcing relationships and that a hypoxic environment stimulates tumor progression and causes an increase in oxygen consumption as tumor cells grow. Compared with the normal environment, hypoxia leads to inhibition of the function of a variety of immune cells. It has been reported that the proliferation and activation of T cells and their effector cells are significantly inhibited by hypoxia, resulting in immune dysfunction [74, 75]. A large number of studies have shown that the growth of B cells and the killing effect of natural killer (NK) cells on tumors are also affected [76]. In addition, various immunosuppressive cells such as TA-MSCs, CAFs, and angiogenic cells co-form conditions conducive to malignant tumor progression under the influence of HIF [77, 78]. Based on the understanding of m^6^A methylation, TME, and hypoxia, we can try to link them more closely with tumors and use them as targets to provide new strategies for the diagnosis and treatment of tumors.

### m^6^A methylation and TME metabolic reprogramming

In TME, metabolic reprogramming has a significant impact on the growth of tumor cells and the function of immune cells [79]. The metabolism of tumor cells is not only regulated by m^6^A methylation, but also closely related to the hypoxia we mentioned in the previous section, and their interaction together affects tumor growth, proliferation, and chemoresistance [20] (Fig. 2).

#### Glucose metabolism

The main route of energy acquisition by tumor cells is glucose metabolism, and abnormal glucose metabolism is the primary characteristic of metabolic reprogramming in TME. Cancer cells take up more glucose and ferment glucose to lactate compared to normal cells, a phenomenon also known as the Warburg effect [80, 81]. In hypoxia, m^6^A methylation promotes the metabolism of glycolysis and enhances metabolic reprogramming, which in turn causes tumor development [82]. Metabolic changes can inhibit the function of various immune cells in TME, such as T cells and NK cells [83]. Thus, glycolytic metabolism has an important role to play in both cellular and humoral immunity [84].

Numerous studies have shown that m^6^A methylation functions to significantly regulate glycolytic reprogramming in tumors. Various proteins affecting m^6^A methylation, such as YTHDF1/2/3, YTHDC1/2, and WTAP can affect the Warburg effect. YTHDF1 promotes glycolysis by up-regulating the expression level of mRNA, thereby accelerating tumor progression [85, 86]. It has been shown that YTHDF3 can destabilize the long noncoding RNA GAS5 and promote CRC progression [87]. YTHDC1 can target miR-30d, a tumor suppressor gene that modifies pancreatic cancer, which in turn inhibits glycolysis and controls the development of pancreatic cancer [88]. As m^6^A writers, METTL3 and WTAP can promote the Walburg effect of tumors, the former can promote both non-small cell lung cancer and breast cancer, and the latter can promote the progression of GC [86, 89, 90]. The presence of FTO promotes the metastasis and spread of HCC, and inhibition of its activity can alter the cell cycle and affect the demethylation of glycolytic pyruvate kinase isoenzyme PKM2 to inhibit tumor development [91].

Multiple lines of evidence show that modification by m^6^A can affect glycolysis, reduce the immune function of T cells, and lead to immune evasion [92]. With these conclusions, we can link metabolic reprogramming to cancer immunotherapy more practically in the future.

#### Lipid metabolism

Lipid metabolism is an important condition for maintaining homeostasis of the intracellular environment and regulating immune responses [79, 93]. Driven by m^6^A methylation and related enzymes, various lipids such as phospholipids and triglycerides are catabolized to produce the energy required by tumor cells and provide nutritional support for the tumor survival environment [94]. Meanwhile, the growth and spread of tumor cells are also affected by lipid metabolism [95]. Numerous studies have shown that m^6^A-mediated reprogramming of lipid metabolism is closely associated with tumor development [96].

It was found that reducing fatty acid content and inhibiting cholesterol esterification effectively enhanced the antitumor immune function of CD8^**+**^ T cells [97]. When the cholesterol content in TME is high, it leads to the decrease of T cells and causes immunosuppression [98]. Fatty acid oxidation (FAO) has also been reported to have an inhibitory effect on the ability of tumors to kill cells and affect antitumor immunity [99]. PRG2 produced by lipid metabolism also exerts inhibitory effects on various immune cells, such as macrophages and NK cells, causing drug resistance and immune evasion [100].

Several researchers have proposed that m^6^A methylation plays a regulatory role in lipid metabolism in TME. It is known that lipid deposition in HCC is regulated by carboxylesterase 2 (CES2), and METTL3 can reduce the level of CES2 and promote the expression of fat complexes and coordinate to deposit lipids [96, 101]. In addition, FTO and ALKBH5 enhanced the expression of multiple regulators in liver tissue [102]. Therefore, knockdown of m^6^A writes METTL3 and m^6^A erasers FTO or ALKBH5 inhibited and promoted lipid accumulation, respectively. FTO has also been demonstrated to be associated with lipid droplet generation in esophageal cancer [103]. In addition, in gliomas, YTHDF2 can regulate cholesterol metabolism and form a suitable living environment for tumor cells [104].

#### Amino acid (AA) metabolism

AA are important factors affecting tumor growth progression and immune regulation. When AA metabolism is abnormal, tumor immunity will inhibit inhibition and cause immune evasion [100]. Methionine metabolism has been reported to be disrupted in the tumor setting, resulting in decreased numbers and immune function of T cells and helping to form an immunosuppressive microenvironment [105, 106]. Similarly, glutamine, an AA, can remodel the TME, while entering the tricarboxylic acid cycle (TCA) after decomposition and transformation, providing a raw material for the production ability [107]. During tumor development, abnormal metabolism and degradation of AA cause changes in glutamine content [108]. When glutamine metabolism is enhanced, TCA can play a stable role in tumor cells, and programmed cell death ligand 1 (PD-L1) expression will be promoted when metabolism is inhibited [107, 109]. In addition, glutamine can also regulate macrophage activation and myeloid-derived suppressor cell (MDSCs) function, and then improve the body’s specific immunity [110, 111]. In TME, the metabolism of arginine and serine inhibits T cell's immune function by affecting the proliferation and cell cycle of T cells, respectively [112, 113].

Reprogramming of AA metabolism mediated by m^6^A methylation significantly impacts tumor initiation and biological properties. It has been found that YTHDF1 can promote glutaminase (GLS) protein synthesis in colon tumors and highly expressed YTHDF1 can lead to the development of drug resistance. Therefore, in the treatment of colon tumors, the combination of antitumor drugs and GLS1 inhibitors can promote the apoptosis of tumor cells [114]. In clear cell renal cell carcinoma (ccRCC), FTO inhibits the presence and expression of von Hippel-Lindau (VHL) tumor suppressors, which limit tumor progression and progression by inhibiting FTO. It was also found by sequencing that SLC1A5, a glutamine transporter target of FTO, could selectively affect the survival and proliferation of tumor cells by stimulating metabolic reprogramming of VHL-deficient ccRCC cells [115].

#### Other metabolism

As an important site for cellular metabolism and acquisition of energy, studies of mitochondrial metabolism are essential to explore the growth of cells in the TME [116]. Studies have shown that the mitochondrial enzyme methylenetetrahydrofolate dehydrogenase-2 (MTHFD2) is highly expressed in ccRCC and promotes HIF-2α expression through m^6^A methylation, leading to tumor development. In mitochondria, hypoxia increases MTHFD2 levels and also enhances HIF-2α expression, a positive feedback phenomenon that accelerates the growth of swollen cells [117]. Another study confirmed that increased or decreased m^6^A methylation, by affecting mitochondrial activity, promotes and inhibits tumor progression, respectively [118, 119].

In addition, it has been pointed out that m^6^A methylation also has a potential impact on a variety of diseases through vitamin metabolism. Vitamin D3 has been reported to be able to treat peritoneal dialysis-related peritoneal injury and function in improving peritoneal fibrosis [120]. Vitamin B12 deficiency causes a decrease in *S*-adenosyl methionine (SAM), affects m^6^A methylation, and presents with a range of neurological symptoms, such as memory impairment and mental decline [121]. However, whether tumors are affected by m^6^A methylation and vitamin metabolism has not yet been clarified.

Notably, some m^6^A-methylated binding proteins have also been found to influence disease progression through glycan metabolism in tumors and certain other diseases. For example, in renal injury, we found that IGF2BP2 could decrease m^6^A modification, suppress METTL3 expression, and delay disease progression [122]. METTL3 promotes CRC progression by activating the m^6^A-GLUT1-mTORC1 axis and is promising to assist in improving treatment outcomes [123].

### m^6^A methylation and TME acidic environment

Lactic acid (LA) is the end product of glycolysis, a precursor of gluconeogenesis, and a key energy source for mitochondrial respiration. Interestingly, LA is also involved in the regulation of TME and epigenetic modifications through histone lactation [124]. Through understanding the Warburg effect, we know that increased glycolytic activity regulated by m^6^A leads to the conversion of the production pyruvate into large amounts of LA, forming acidic TME and affecting the growth of tumor cells [125] (Fig. 2). ALKBH5 was found to improve levels of m^6^A and RNA stability by targeting Mct4, a key enzyme that promotes rapid LA plasma membrane transport [16]. In addition, increased LA in TME upregulated METTL3 in tumor-infiltrating myeloid cells (TIMs) and enhanced Janus kinase 1(JAK1) protein translation efficiency and subsequent transcription activator 3(STAT3) phosphorylation via the m^6^A-YTHDF1 axis in CRC [126]. It has been reported that the LA sensor GPR81 is the LA receptor highly expressed in tumor cells, and high expression of GPR81 inhibits the immune effects of T cells and dendritic cells (DCs) and causes immune evasion [127]. LA secretion leads to an increase in MDSCs and T regulatory cells (Tregs), causing a decrease in the activity of NK cells and T lymphocytes and affecting the maturation of DCs [128–130].

GPR81 is highly expressed in breast cancer and supports tumor cell growth through autocrine effects, and down-regulation of GPR81 can inhibit breast cancer progression [131]. In lung cancer, expression of programmed cell death protein 1 (PD-1) and PD-L1, a negative immune regulatory pathway, was also found to be upregulated when LA content was increased. Inhibition of LA synthesis inhibits PD-1 or PD-L1 protein levels and function [132]. Thus, silencing GPR81 signaling could facilitate immunotherapy in cancer.

Increasing evidence shows that LA can promote tumor development, metastasis, and resistance, so some drugs can be tried to inhibit the production and transport of LA, combined with immune drugs in the clinical treatment of tumors to improve tumor efficacy [133].

### m^6^A methylation and TME immunosuppression

A large number of studies have shown that m^6^A methylation not only directly affects the immune response of tumor cells, but also can produce a large number of metabolites through metabolic reprogramming to affect the immune response, causing highly immunosuppressive TME, leading to immune evasion [134, 135] (Fig. 2). In a variety of tumors, high expression of PD-L1 is found, causing T-cell apoptosis by binding to the PD-1 receptor and promoting immune evasion [136]. In breast cancer, METTL3 expression increased PD-L1 stability and expression, and METTL3/IGF2BP3 knockdown significantly enhanced the immune response [137]. YTHDC1 has been shown to promote enhanced cyclization of circlGF2BP3 by METTL3 and increase PD-L1 expression in tumor cells [138]. In addition, LA accumulation in tumor cells can also improve immunosuppressive ability by promoting the expression of METTL3 [126]. Notably, METTL14 also can modulate PD-L1 levels. In cholangiocarcinoma, METTL14 induced the expression of seven in absentia homolog 2(Siah2) in cholangiocarcinoma, which in turn promoted PD-L1 expression levels in cholangiocarcinoma [139]. In HCC, lipopolysaccharide (LPS) increases METTL14 levels and exerts its regulatory effect on PD-L1, while it is important in mediating immune evasion [140]. ALKBH5 has been reported to maintain PD-L1 expression through the ALKBH5-PD-L1 regulatory axis in intrahepatic cholangiocarcinoma while inhibiting T-cell growth and infiltration [141]. Similarly, in head and neck squamous cell carcinoma (HNSCC), the level and function of NK cells are also inhibited by ALKBH5, mediating immunosuppressive TME and promoting tumor growth and progression [142]. Another study confirmed that in prostate cancer, inhibition of glutamine can cause epigenetic modifications, death of cancer stem cells (CSCs), and improve the sensitivity of radiation therapy [143].

The above evidence confirms that m^6^A methylation and various metabolites can cause TME immunosuppression and immune evasion by regulating PD-L1 levels and affecting various immune cells, providing a new angle to solve the difficulties of cancer immunotherapy.

## m^6^A methylation and cells in TME

### Immune cells

Immune cells play an important role in the process of resisting and inhibiting tumor cells, such as T cells, B cells, NK cells, and DCs, and inhibiting the activation, proliferation, and migration of these cells can lead to immunosuppressive TME and cause tumor immune escape [128, 144].

Numerous studies have confirmed that m^6^A methylation promotes immune evasion by inhibiting the differentiation and function of T cells by affecting the expression of transcripts and glycolytic metabolism [92, 145]. In breast cancer, high expression of METTL3 and IGF2BP3 maintains PD-L1 mRNA stability, promotes T cell senescence, and evades immune surveillance [137]. In the absence of METTL3, m^6^A methylation has been reported to protect T cell proliferation by maintaining T cell homeostasis through protection of the JAK-STAT signaling pathway [146]. HIF-1α mediates VHL regulation in T follicular helper (Tfh) cells, and when VHL is decreased, glycolysis and epigenetic modifications are promoted, resulting in reduced numbers of mature T cells [145]. FTO activates transcription factors by mediating m^6^A demethylation, increases glycolytic metabolic activity, weakens the function of CD8^+^ T cells, and promotes tumor growth [92]. Meanwhile, Tfh cells can enhance the function of CD8^+^ T cells, exert antitumor immunity, and improve the effect of immunotherapy [147, 148]. Some researchers have found that METTL3-mediated m^6^A methylation can not only affect the differentiation of T cells but also maintain the inhibitory effect of Tregs on the tumor-killing function of CD8^+^ T cells.

Notably, B cell development and function are also regulated by m^6^A methylation. YTHDF2 limits early B cell development and proliferation and impacts immune responses [149]. In lung adenocarcinoma (LUAD), nucleophosmin 1 (NPM1) impacts B and NK cell survival through glycolytic metabolism and YTHDF2-mediated m^6^A methylation [150].

Macrophages can phagocytose and eliminate cell debris, tumor cells, and other harmful substances, but also activate the immune system and regulate immune response [151, 152]. Macrophages can be divided into two immunologically distinct subpopulations, classically activated macrophages (M1 phenotype) and alternatively activated macrophages (M2 phenotype) [152]. M1 not only secretes pro-inflammatory mediators but also has high antigen extraction and tumoricidal effects [153, 154]. M2 plays an immunosuppressive role, which facilitates the growth of tumor cells and promotes tumor progression [25]. Under certain conditions, macrophages transform into tumor-associated macrophages (TAM) in the TME and have functions similar to M2, causing the spread and immune evasion of tumor cells [152]. Gu Y, and her companions found that TAM caused immunosuppressive TME by expressing inhibitory receptors leading to T-cell reduction [20]. Several researchers have proposed that m^6^A methylation mediates polarization in macrophages and impacts TME homeostasis. METTL3-mediated methylation of m^6^A improves tumor killing by macrophages by enhancing the ability of TAM to polarize toward M1 [153]. Similarly, in animal experiments, it was found that when METTL3 expression was suppressed in mice, M2 was markedly stimulated and M1 activation was suppressed [155]. Du et al. showed that deletion of METTL14 or YTHDF1 resulted in macrophage defects as evidenced by hyperactivation and high inflammation [156]. FTO depletion suppressed nuclear factor κ-light-chain enhancer of activated B cells (NF-κB) pathway and STAT1/6 expression and restricted M1 and M2 polarization [157]. Furthermore, in inflammatory responses, LPS-mediated IGF2BP2 resulted in attenuated M1 phenotype of macrophages, thereby suppressing inflammatory responses [158].

DCs, which can antigen processing and activate naive T cells, are the strongest antigen-presenting cells (APC) and can elicit antitumor immune responses [159]. Increased expression of DCs promotes immune surveillance and inhibits immune evasion [160]. Many studies have found aberrant modification of m^6^A methylation in DCs from TME. It has been reported that YTHDF1 binds to transcripts encoding lysosomes, enhances the translation and expression of lysosomal proteases, affects the activation of CD8^+^ T cells and DCs, and inhibits antigen presentation. Notably, silencing YTHDF1 enhanced the efficacy of PD-L1 anti-immunotherapy [161]. Interestingly, another study found that the knockdown of YTHDF1 in GC caused the accumulation of mature DCs and promoted CD4^+^ and CD8^+^ T cell infiltration, perhaps favoring antitumor immune sensitivity [162]. CC-chemokine receptor 7 (CCR7) affects HIF-1α activity and inhibits glycolytic metabolism and migration of DCs. In addition, CCR7 can also act on m^6^A methylation and mediate the expression of the long noncoding RNA lnc-Dpf3, which binds HIF-1α and hinders DCs migration [163]. YTHDF2 causes loss of DCs function by affecting CCR7-mediated m^6^A methylation. There is evidence that vaccination with DCs has a significant adjuvant effect on immunotherapy of tumors [164]. These studies suggest that m^6^A methylation has a significant impact on the antitumor immunity of DCs and can serve as a critical mechanism for immunotherapy.

NK cells have cytotoxic effects and can directly kill target cells and control tumor progression by inhibiting the proliferation and metastasis of tumor cells. In addition, a variety of cytokines, including interferons, can be produced, thereby regulating immune responses [165, 166]. Therefore, targeting NK cells is a novel therapeutic approach in immune cells of tumors [167, 168]. It has been found that m^6^A methylation affects NK cells. YTHDF2 has been reported to maintain NK cell homeostasis and exert immune effects, and reduced expression levels of YTHDF2 inhibit the antitumor effects of NK cells [169]. Another study showed that promoting the expression of METTL3 enhanced the immune surveillance of NK cells, and METTL3 knockdown inhibited the response of NK cells to interleukin-15, affected the homeostasis and tumoricidal function of NK cells, and promoted tumor growth [170].

It has been found that eosinophils, basophils, and neutrophils, these three granulocytes can respond to various stimuli including inflammation and tumors [171, 172]. A large body of evidence suggests that enhancing granulocyte activity can promote antitumor effects [173, 174]. However, m^6^A methylation has a role in affecting granulocyte function. In ccRCC, it was found that the expression level of YTHDF2 significantly affected the degree of neutrophil infiltration, thereby affecting the survival of patients [175]. The expression of METTL14 and ZC3H13 was positively correlated with the expression of neutrophils in breast cancer [176]. Eosinophils have also been reported to be regulated by CD4^+^ T cells in HNSCC, and FTO and ALKBH5 have a reverse effect on survival and immune infiltration [177]. Based on these studies, granulocytes as therapeutic targets for tumors could assist in improving the efficacy of immunotherapy and inhibiting immune evasion and tumor progression. MDSCs are immune cells with immunosuppressive effects in TME and can differentiate into various immune cells, such as macrophages under physiological conditions, but they proliferate significantly in pathological environments including tumors, and play a strong role in immunoregulatory processes and inhibit immune responses [178, 179]. Studies have confirmed that m^6^A methylation affects the function of MDSCs themselves as well as the regulation of immune responses. For example, METTL3 has been found to stimulate MDSCs differentiation in cervical cancer, and the expression of the two is positively correlated, promoting tumor progression and affecting prognosis [180]. In addition, ALKBH5 inhibits immune infiltration and accumulation of MDSCs thereby regulating immune responses. In melanoma, ALKBH5 suppressed immunity and promoted immune evasion by affecting Tregs and MDSCs, while ALKBH5 knockdown significantly suppressed the expression of Tregs and MDSCs and decreased immune suppression [16]. Monocarboxylate transporter 4 reduces lactate concentration by targeting ALKBH5, thereby affecting the content of Tregs and MDSCs [78]. In HNSCC, m^6^A methylation was found to be significantly associated with the infiltration of a variety of immune cells, but no mention was made of the study of MDSCs, which could be used as a research direction in the future to provide new strategies for immunotherapy of HNSCC [177].

### Nonimmune cells

Mesenchymal stem cells (MSCs) interact with the TME and are complex and able to modulate multiple immune responses in the TME. MSCs can not only control tumor progression by activating immune responses in an APC manner but also promote immune evasion by inhibiting the polarization of various immune cells [181]. Currently, most of the effects of m^6^A methylation on MSCs have focused on metastatic sites of tumors, while studies on primary TME have been virtually absent. Bone marrow is rich in blood vessels and nutrients, so it is one of the common metastatic sites of tumors. It has been reported that m^6^A methylation regulates the differentiation of bone marrow mesenchymal stem cells (BM-MSCs) into osteoblasts. METTL3 expression was found to be higher in BM-MSCs during osteogenic induction, and METTL3 knockdown resulted in impaired BM-MSCs differentiation, which may be associated with decreased phosphorylation in the AKT signaling pathway [182, 183]. Moreover, m^6^A methylation acts on MSCs differentiation by affecting the translation of parathyroid hormone receptor-1 (Pth1r) [182]. In TME, MSCs have been shown to transform into TA-MSCs in response to certain cytokines and promote the growth of tumor cells. In pancreatic cancer, up-regulation of TA-MSCs enhances macrophage migration inhibitory factor (MIF) expression and promotes the malignant biological behavior of tumors by increasing levels of FTO. Inhibition of MIF expression then reduces the level of FTO to exert a tumor suppressor effect [184]. The relationship between m^6^A methylation and MSCs in TME and more clear mechanisms need to be further studied, and based on the different effects of MSCs on tumors, it may be possible to use targeted MSCs drugs to make treatment beneficial to enhance immunity and inhibit immune evasion.

CAFs are important components of TME that promote tumor progression and immune evasion, not only enhancing the recruitment of myeloid-derived suppressor cells but also promoting the transformation of various immune cells such as macrophages [185]. Activation of CAFs is induced by several growth factors including transforming factor β and fibroblast growth factor 2 [186]. Notably, stromal cell-derived factor 1 (SDF-1) secreted by CAFs can both enhance the ability of angiogenesis by promoting the recruitment of endothelial progenitor cells (EPCs) but also affect C-X-C chemokine receptor (CXCR) 4 to exert a tumor-promoting effect [187]. In ovarian cancer, enhanced expression of C-X-C motif ligand (CXCL) 14 in CAFs impacts glycolysis and promotes tumor metastasis [188]. CAFs can also transform cancer cells into CSCs through epithelial-mesenchymal transition (EMT) and promote tumor malignancy and immune tolerance [189–191]. However, few existing studies have focused on the interrelationship between m^6^A methylation and CAFs. Only in recent years, researchers have found that in 3T3-L1 cells, the process of adipogenesis mediated by fibroblasts is affected by m^6^A modification, and FTO and YTHDF2 play a significant role [192]. Considering the multiple ways in which CAFs mediate tumor invasion, the role of m^6^A modification in the secretion of functional factors by CAFs could be investigated as a potential target for cancer therapy in the future.

Angiogenic cells are indispensable components in the development of tumors and provide nutritional needs and metabolic sites of tumor cells. As tumors progress, normal blood vessels fail to meet their needs, and multiple components of the TME re-synthesize other blood circulation [193]. Tumor angiogenesis can not only affect the function of cells in TME but also inhibit tumor cell death. In addition, vascular endothelial cells (VECs) also provide conditions for tumor angiogenesis to play an important role in mediating the growth and metastasis of tumor cells, which also inhibits the production of pericytes and vascular smooth muscle cells (VSMCs) [194]. In turn, various cells, such as CAFs and TAM in TME can also secrete factors, such as cytokines, vascular endothelial growth factor (VEGF), CXCL12, and interleukins to accelerate tumor angiogenesis [195–197]. A large number of studies have shown that m^6^A methylation is involved in the regulation of tumor angiogenesis. It has been reported that forms of IGF2BP2 exosomes migrate to endothelial cells, promote angiogenesis in LUAD, cause tumor invasion and poor prognosis, and IGF2BP2 knockdown inhibits tumor metastasis and angiogenesis [198]. Another study also confirmed that YTHDC2-mediated m^6^A methylation enhances the ability of angiogenesis in lung cancer by promoting the expression of vascular endothelial growth factor A (VEGFA) [199]. In HCC, METTL3 expression was found to positively correlate with angiogenesis and significantly affect angiogenesis, and in addition, m^6^A methylation levels also affected HCC stage and prognosis, while negatively regulating tumor response to anti-angiogenic drugs [200, 201]. In tongue squamous cell carcinoma (TSCC), METTL14 enhanced VEGFA expression and promoted TSCC development and angiogenesis by inhibiting basic leucine zipper ATF-like transcription factor 2 (BATF2) [202]. Studies have shown that METTL3 knockdown can directly affect the inhibition of angiogenesis and also reduce angiogenesis by affecting the Wnt pathway. In addition, YTHDF1 expression also affects angiogenesis by mediating Wnt signaling [203]. Furthermore, evidence has confirmed that IGF2BP2/3 mediates the pro-angiogenic effects of METTL3 in CRC and, together, promotes tumor progression [204].

**Fig. 3:**
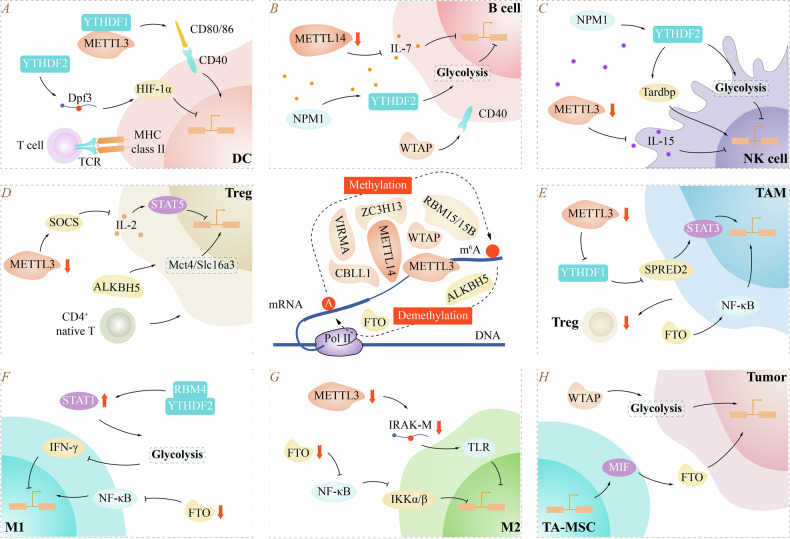
m^6^A modification in various cells. m^6^A methylation is regulated by the m^6^A methyltransferase complex (writers), m^6^A demethylase (erasers), and m^6^A binding protein (readers). Regulators associated with m^6^A methylation affects activation and function of DC cells (**A**), function of B cell and Treg (**B**, **D**), homeostasis and function of NK cell (**C**), polarization and plasticity of TAM (**E**), M1 and M2 (**F**, **G**), differentiation and function of TA-MSC (**H**).

Increasing evidence suggests that m^6^A methylation can remodel the TME, affect the development and function of various cells, mediate immune escape, regulate immune responses, and affect tumor progression (Fig. 3).

## Role of m^6^A methylation in TME

### Regulation of the cell cycle

The cell cycle is divided into four phases: G1, S, G2, and M phase, which is the complete embodiment of cell division. The occurrence and development of tumors are inseparable from cell division. It has been reported that cell cycle arrest in the G1 phase mediated by small interfering RNA (siRNA) has the effect of preventing tumor progression [205]. A large number of aberrant cell cycle pathways are found in gastrointestinal malignancies, leading to abnormal proliferation of tumor cells [206]. Increasing evidence suggests that m^6^A methylation can regulate the cell cycle, thereby promoting tumor development. Decreased expression of WTAP has been shown to disrupt the transforming growth factor β (TGF-β) signaling pathway, inhibit cell cycle arrest, and promote tumor proliferation [207]. METTL3 expression in cervical cancer leads to accelerated cell cycle progression and tumor proliferation [208]. In addition, inhibition of METTL3 expression has also been found to result in abnormal cell cycle activity, neuronal death, and memory impairment in Alzheimer’s disease [209]. YTHDF1 promotes the growth of tumor cells in LUAD by enhancing the expression of proteins in the cell cycle and affects the prognosis and stage of tumors [210]. To date, a large body of evidence has established that IGF2BP protein has been found to affect the cell cycle in different ways in a variety of tumors and then have an impact on the development of tumors, such as CRC, ccRCC, thyroid cancer, and endometrial cancer [211–214].

### Involved in apoptosis

Apoptosis is a major pathway of cell death in a variety of cells, including cancer cells. Apoptosis has dual effects on tumors, both inhibitory and promoting properties [215, 216]. Studies have shown that apoptosis not only affects cancer cells but also affects various cellular components in TME such as endothelial cells [217]. Interestingly, m^6^A methylation has also been found to be associated with apoptosis [218]. For example, enhancing METTL3 expression or knocking down ALKBH5 expression in cardiomyocytes promotes apoptosis in animal experiments [39]. Knockdown of FTO promoted apoptosis in both leukemia and LUSC [35, 219]. Recent evidence confirms that METTL3 promotes PCBP2 stability, inhibits apoptosis, and accelerates tumor metastasis in gliomas [220]. Inhibition of p53 mRNA m^6^A methylation by inhibiting METTL3 expression enhances HCC cancer cell apoptosis and tumor therapeutic efficacy [221]. In addition, some researchers have proposed that hypoxia and metabolism also have a role in affecting apoptosis. Specifically, hypoxia can inhibit apoptosis not only by directly activating glycolysis but also cause tumor evasion of apoptosis by affecting EMT/ALKBH5 expression of EMT and angiogenesis-related transcripts [222–224]. In CRC, mitochondrial metabolic reprogramming in cancer cells is inhibited by apoptosis caused by regulation of the RNA-binding protein RALY, while METTL3 can affect tumor development by targeting RALY [225].

### Regulated autophagy

Autophagy is a type II programmed cell death mode that has both promoting and inhibiting effects on tumors at different stages. Specifically, in the pre-neoplastic stage, autophagy functions to delay tumor progression through its survival pathways and quality control mechanisms. In the later stage of the tumor, autophagy promotes the development of the tumor and the malignant biological function of the tumor by promoting metabolic reprogramming [226, 227]. For example, researchers have found that autophagy has two different roles in oral squamous cell carcinoma. On the one hand, autophagy plays a protective role by inhibiting cancer cell death, thereby enhancing the growth ability of tumors [228]. Moreover, inhibition of autophagy by using 3-methyladenine and chloroquine promotes apoptosis in oral squamous cell carcinoma and enhances tumor sensitivity to drugs [229]. On the other hand, autophagy plays a role in inhibiting tumor invasion and metastasis by inhibiting NF-κB pathway and AKT/mTOR/ NF-κB pathway [230, 231]. Evidence suggests that hypoxic environment promotes tumor growth by regulating autophagy. In glioblastoma, PAK1 (p21 activated kinase 1) in the hypoxic environment accelerates the proliferation of tumor cells by mediating autophagy [232]. Increasing evidence supports the theory that autophagy-related mechanisms behind tumor progression are associated with m^6^A methylation. Numerous studies have shown that m^6^A methylation affects tumor development mediated by affecting autophagy. In HCC, YTHDF1 binds to mRNA to enhance the expression of autophagy-related genes and increase the possibility of tumorigenesis, while knockdown of YTHDF1 inhibits autophagy and delays tumor progression [66]. In hypoxia, inhibition of METTL3 expression caused therapeutic resistance of HCC to sorafenib, a drug, while triggering autophagy as a mechanism [233]. Pretreatment with omeprazole targets FTO in GC inhibits the expression of the latter, and improves the efficacy of antineoplastic drugs in GC by inhibiting autophagy [234]. By downregulating the expression of LINRIS, a prognostic biomarker in CRC, K139 ubiquitination of IGF2BP2 is inhibited, avoiding its degradation via the autophagolysosomal pathway, thereby inhibiting the proliferation of cancer cells [235].

### Regulated immune checkpoints

Blocking immune checkpoints has become one of the important means in cancer therapy, for example, PD-1 and PD-L1 are common immune checkpoint molecules in TME. In clinical practice, the application of antitumor drugs such as anti-PD-1 and anti-PD-L1 provides great help for the treatment of tumors. Studies have shown that m^6^A methylation has some effect on certain immune checkpoints. For example, promoting FTO expression increases PD-L1 expression in colon cancer, whereas the knockdown of FTO suppresses PD-L1 expression levels [236]. Notably, in leukemia, inhibition or reduction of FTO expression blocks leukocyte immunoglobulin-like receptor B4, an immune checkpoint gene expression, inhibits the self-renewal ability of stem cells, while enhancing leukemia sensitivity to drugs and preventing the development of immune evasion [237]. Knockdown of METTL3 and METTL14 was found to improve the efficacy of anti-PD-1 drugs in both CRC and melanoma tumors [238]. In addition, reducing the expression of FTO promotes the expression level of PD-1, thereby improving melanoma resistance to immunotherapy [239]. Interestingly, knocking down ALKBH5 affects the accumulation of multiple immune cells and transcriptional expression of tumor cells and similarly improves the sensitivity of melanoma to anti-PD-1 drug therapy [16]. A recent study showed that inhibition of YTHDF1 expression delayed tumor progression by increasing CD8^**+**^ T cell content or in combination with the use of anti-PD-1 drugs [240].

### Associated with therapeutic resistance

During the treatment of tumors, resistance to various treatment modalities, such as chemotherapy and radiation therapy often occurs. Therefore, to improve the therapeutic effect and block the occurrence of this phenomenon [241]. There is a large body of literature reporting the mechanisms involved in therapeutic resistance. Evidence has shown that YTHDF1 reduces proliferation and metastasis in non-small cell lung cancer by regulating the translational efficiency of multiple proteins. Knockdown of YTHDF1 slows the development of LUAD, but, at the same time, leads to tumor resistance to cisplatin and adaptation to a hypoxic environment [242]. Interestingly, in CRC, YTHDF1 promotes cancer cell proliferation and metastasis, and enhancing YTHDF1 expression inhibits tumor sensitivity to cisplatin [243]. Alternatively, YTHDF1 contributes to the development of cisplatin resistance by affecting metabolic reprogramming [114]. This suggests that YTHDF1 may have different effects in the treatment of different tumors, and may be used as a target in the future to improve the effect of cancer treatment according to its role in different tumors. Increasing estrogen receptor-γ levels by promoting METTL3 expression can accelerate fatty acid oxidation and metabolic reprogramming, thereby affecting tumor progression and chemoresistance [244]. Elevated expression levels of METTL3 have been found to contribute to tumor development by promoting adenylate kinase-4 expression in breast cancer while causing tamoxifen treatment resistance [245]. In addition, it has been found in breast cancer that FTO can inhibit the expression of β-catenin, which leads to tumor chemoradiation resistance [246]. In glioma, it was found that ALKBH5 not only affected tumor invasion ability, but also mediated tumor radioresistance, and the knockdown of ALKBH5 could play an active role in the treatment of glioma and improve its sensitivity to radiation therapy [247]. By inhibiting METTL3 expression in pancreatic cancer, tumor sensitivity to a variety of chemotherapeutic agents and radiation therapy can be improved [248].

## Effect of m^6^A methylation on pre-metastatic niche (PMN) and metastatic niche (MN)

Tumor cells at the primary site enter the circulation after destroying the surrounding tissue, and approximately 0.01% of these cells can survive at distant sites. Subsequently, these tumor cells develop a proliferative state and constitute tumor metastasis [249, 250]. Metastasis is one of the biological characteristics of tumors, and by metastasis, tumors develop from local disease to systemic disease, so metastasis is a key lethal factor in cancer patients. TME is a background in the development of metastasis and provides the necessary support for the survival of tumor cells.

PMN and MN are key processes to promote metastasis, which constitute a microenvironment conducive to tumor growth for the primary tumor on the site of subsequent metastasis and play an important role in the progression of metastasis. Primary tumor-derived factors, bone marrow-derived cells, and multiple changes in stromal components are three important reasons contributing to the PMN establishment. Immunosuppression, inflammation, high angiogenesis and vascular permeability, lymph angiogenesis, organ tropism, and reprogramming are six important features of PMN that determine the properties of PMN [251].

Many researchers have found that PMN, MN, and TME play an important role in driving tumor metastasis.

Evidence has shown that autophagy in TME can promote angiogenesis and PMN formation in distant metastatic sites of tumors by affecting CAFs [252, 253].

Extracellular vesicles (EVs) are lipid bilayer membrane nanostructures released from cells, which are one of the important components in TME and function to mediate cellular communication and participate in a variety of pathophysiological processes. Small extracellular vesicles (sEVs) are a subtype of EVs involved in cell-to-cell signaling communication in the TME [254]. It has been found that sEVs mediate metabolic reprogramming by affecting various cells in TME, such as tumor cells and CAFs, Tregs, MDSC, and NK cells, forming an acidic microenvironment conducive to PMN formation and promoting tumor metastasis [255].

In addition, tumor cells and various cells in TME can secrete subsets of EVs, i.e., exosomes, which promote tumor progression by promoting angiogenesis, altering TME, forming PMN, and inducing immunosuppression [256].

Evidence suggests that m^6^A is also associated with PMN formation. Above, we mentioned that CAFs are one of the important components of TME, and the researchers found that in breast cancer, the long noncoding small nucleolar RNA host gene (Inc-SNHG5) can bind to IGF2BP2 and promote the stability and expression level of ZNF281, a zinc finger factor in CAFs, which induces the formation of PMN [257]. Interestingly, recent animal research also found that in melanoma, inhibiting IGF2BP1 expression affected CD45 levels and then PMN formation, significantly reducing the probability of EVs-mediated lung metastasis in melanoma [258].

In summary, perhaps we can control the occurrence of tumor metastasis by limiting PMN formation, which can not only improve the quality of life in cancer patients but also significantly prolong their survival.

## Potential clinical applications of m^6^A methylation

### Role of m^6^A methylation in diagnosis and prognosis of tumors

Definite diagnosis of tumors is of great significance for the treatment and prognosis of tumors, which can not only help to improve the therapeutic effect, improve the prognosis, but also improve the survival rate of patients. Numerous studies have found that m^6^A methylation and its modulators play an important role in the diagnosis and prognosis of tumors [9, 259]. For example, down-regulation of METTL14 is found in metastatic HCC, and this change can be used to determine the prognosis of the tumor [260]. Evidence suggests that METTL14 and METTL3 have different implications for poor tumor prognosis in CRC. Knockdown of the former leads to poor prognosis of tumors by decreasing the expression of YTHDF2 [13, 261]. In contrast, inhibition of the latter expression leads to improved tumor prognosis [262]. A significant increase in m^6^A was observed in non-small cell lung cancer, and the level of m^6^A was significantly higher in LUSC than in LUAD and showed high sensitivity and specificity when helping to diagnose LUSC [263]. Mutations in the TP53 gene are one of the main factors mediating the development of LUAD, and it has been found that both YTHDF1/2 and WTAP promote expression through genetic mutations [264]. In addition, various m^6^A methylation modulators such as METTL3, FTO, and IGF2BP have been found to have some relationship with tumor prognosis [27]. In esophageal cancer, the expression of METTL3, IGF2BP3, and WTAP is increased, and all of them have been confirmed to be related to the extent of tumor invasion, lymph node and adjacent organ invasion, distant metastasis, and other criteria used to identify the stage, and by observing the m^6^A methylation expression level to help the early diagnosis of tumors and determine the prognosis of tumors [265–267]. At present, because the expression of m^6^A methylation is different in different humans and the means of examination are not complete, its help in practical clinical practice is still limited. However, this can be used as a research direction in the future, with the help of single-cell sequencing technology and other more advanced means that may make it better applied in clinical practice.

### Therapeutic strategies targeting m^6^A methylation

A large number of studies have shown that the use of m^6^A methylation modulators plays an important role in the treatment of tumors by affecting tumor growth, proliferation, and metastasis. For example, inhibition of FTO expression using FB23 and FB23-2 affects cell cycle and apoptosis in acute myeloid leukemia (AML), which can significantly damage the growth and proliferation of cancer cells, and then achieve the purpose of cancer therapy [268]. Recent studies have shown that CS1 and CS2, two newly identified inhibitors of FTO, have a more significant effect in reducing cancer cell viability compared to FB23 and FB23-2 [237]. Interestingly, as an inhibitor of METTL3, STM2457 also had a therapeutic effect in mice with AML, improving the survival of mice by reducing the number of leukemic stem cells and tumor cells [269]. In addition, IGF2BP1 inhibitors can also be effective in the treatment of leukemia by affecting certain regulators and regulatory enzymes [270]. Similarly, inhibition of IGF2BP1 inhibited cell proliferation, thereby slowing ovarian cancer and melanoma progression [271]. In glioblastoma, inhibition of METTL3 and METTL14 expression promotes the development of glioblastoma, and up-regulation of METTL3 or inhibition of FTO expression through MA2 delays tumor growth [272]. In the clinic, some drugs exert antitumor effects by targeting m^6^A methylation. For example, berberine inhibits IGF2BP3 expression, and hampers the normal process of the cell cycle by affecting the PI3K/AKT pathway, which in turn inhibits cancer cell proliferation in CRC [273]. In CRC and HCC, both benzamide benzoic acid and urea-thiophene compounds could inhibit tumor growth by downregulating IGF2BP2 [274]. Inhibition of IGF2BP3 expression by using JQ1 has the effect of slowing the growth and migration of cancer cells in Ewing sarcoma and improving poor prognosis [275].

### Effect of m^6^A methylation in combination therapy

To date, there are various treatments for tumors, including chemotherapy, radiation therapy, and targeted therapy. However, it is more difficult that tumor progression leads to the development of therapeutic resistance. Based on the study of m^6^A methylation, this can be used as a target in combination with other tumor treatments to achieve better therapeutic goals. As mentioned above, metabolic reprogramming, such as glycolysis and mitochondrial metabolism in TME is involved in tumor development and growth, so targeting these metabolic pathways may be able to help tumor treatment. Researchers have confirmed that bis-2- (5-phenylacetamido-1,3,4-thiadiazol-2-yl) ethyl thioether inhibits the development of tumors by inhibiting glutaminase to affect glutamate metabolism, and its combination with poly (ADP-ribose) polymerases inhibitors can better function in the treatment of tumors [276]. For example, inhibition of METTL3 expression combined with the glycolytic inhibitor 2-deoxyglucose has a role in delaying tumor progression in HCC [277]. Similarly, in seminomas, tumor sensitivity to cisplatin was improved by inhibiting METTL3 and autophagy, improving therapeutic efficacy [278]. IGF2BP3 and HIF-1α together affect cancer cell metastasis and neovascularization, while inhibiting their expression better inhibits adverse factors that accelerate tumor progression [69]. In addition, combined blockade of PD-L1 checkpoint and expression of YTHDF1, FTO, and METTL3/14 was able to improve the significant effect in the treatment of CRC and leukemia [161, 237, 238].

## Conclusions and perspectives

Based on the current high incidence and low cure rate of tumors, it is important to understand the mechanisms that influence tumor development as well as treatment and prognosis.

In this paper, we introduce the m^6^A methylation-related proteins closely related to tumors and the characteristic changes of TME, analyze the relationship between m^6^A methylation, TME, and tumors and the existing mechanisms and tumor treatment patterns, and elaborate that complex and variable m^6^A methylation is an important factor affecting TME and tumor development. In addition, we also present the effects of m^6^A methylation, TME, and PMN on tumor metastasis for the first time.

However, the disadvantage is that because the understanding of the regulatory mechanism of TME is still lacking in studies so far, the mutual influence and crosstalk in TME cannot be fully understood, but hypoxia, metabolic reprogramming, acidic environment, and immunosuppression have been proposed in TME, respectively, without linking them to each other. In the future, we can understand the complex crosstalk of TME by deeply exploring ways in which multiple cell populations communicate in different times and spaces.

In summary, m^6^A methylation has a definite value in the diagnosis of tumors and can be used as a tumor marker to predict the occurrence and development of tumors. In addition, because there will be different m^6^A modification patterns in different tumors, m^6^A affects tumor growth, proliferation, and metastasis, so targeted m^6^A antitumor therapy is a promising treatment in different tumor tissues or cells. As the survival background of tumor cells, treatment strategies for TME can also provide new ideas for treating tumors. Therefore, combining therapies targeting m^6^A methylation, metabolic reprogramming, hypoxia, and immunosuppression may better address the complexity of tumorigenesis factors, multiple difficulties in cancer treatment, and complex mechanisms in TME.

In future studies, we can also further investigate the regulatory mechanisms between m^6^A, TME, and tumors and reveal more cancer treatment strategies. As a potential target for antitumor therapy, it is expected that these studies will also help reduce the efficacy and side effects of individualized precision medicine for cancer patients and provide more new ideas for diagnosis.
